# Supplementary material for: Integrating genomic structural equation modeling and experimental validation to unravel the genetic basis of male genital lichen sclerosus
Source: Front Immunol. 2026 Jul 16;17:1857945. doi: 10.3389/fimmu.2026.1857945 (PMC13435250; doi:10.3389/fimmu.2026.1857945)
Supplement: Supplementary file 6 [file SupplementaryFile5.zip › result/fuma_PSYCH_factor_sCCA1_focus.chr1.chr1.44969183.46899501.pop1.pdf]

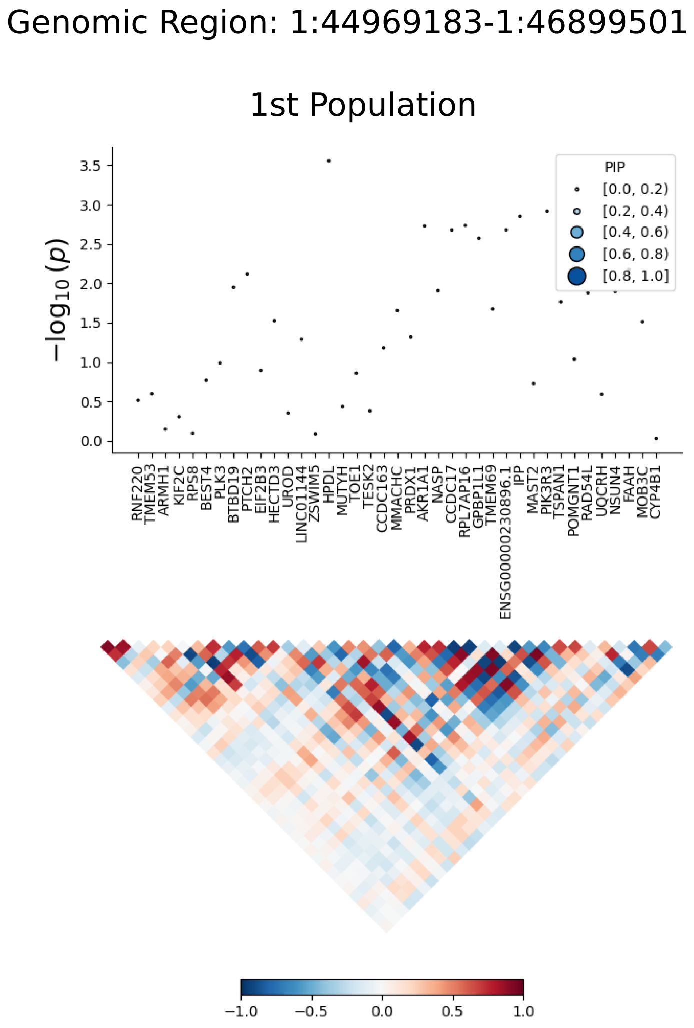
Genomic Region: 1:44969183-1:46899501
1st Population
$-\log_{10}(p)$
0.0
0.5
1.0
1.5
2.0
2.5
3.0
3.5
PIP
[0.0, 0.2)
[0.2, 0.4)
[0.4, 0.6)
[0.6, 0.8)
[0.8, 1.0]
RNF220
TMEM53
ARMH1
KIF2C
RPS8
BEST4
PLK3
BTBD19
PTCH2
EIF2B3
HECTD3
UROD
LINC01144
ZSWIM5
HPDL
MUTYH
TOE1
TESK2
CCDC163
MMACHC
PRDX1
AKR1A1
NASP
CCDC17
RPL7AP16
GPBP1L1
TMEM69
ENSG00000230896.1
IPP
MAST2
PIK3R3
TSPAN1
POMGNT1
RAD54L
UQCRH
NSUN4
FAAH
MOB3C
CYP4B1
−1.0
−0.5
0.0
0.5
1.0
